# Identification of Factors Necessary for Enabling Technology-Based Dietary Record Surveys: A Qualitative Focus Group Interview with Japanese Dietitians

**DOI:** 10.3390/nu14204357

**Published:** 2022-10-18

**Authors:** Yuko Tousen, Chifumi Shimomura, Ai Yasudomi, Yukie Kaneda, Nanako Nishiwaki, Mayumi Fujita, Hiroko Oya, Toshiro Kobori, Masuko Kobori, Hidemi Takimoto

**Affiliations:** 1 Department of Food Function and Labeling, National Institute of Health and Nutrition, National Institutes of Biomedical Innovation, Health and Nutrition, 1-23-1 Toyama, Shinjuku-ku, Tokyo 162-8636, Japan; 2 Department of Nutritional Epidemiology and Shokuiku, National Institute of Health and Nutrition, National Institutes of Biomedical Innovation, Health and Nutrition, 1-23-1 Toyama, Shinjuku-ku, Tokyo 162-8636, Japan; 3 Department of Research and Development, Healthcare Systems Co., Ltd., 1-14-8 Shirakane, Showa-ku, Nagoya 466-0058, Aichi, Japan; 4 Institute of Food Research, National Agriculture and Food Research Organization (NARO), 2-1-12 Kannondai, Tsukuba 305-8642, Ibaraki, Japan

**Keywords:** dietary record, information and communication technology, dietary assessment, focus group interview, non-face-to-face

## Abstract

Weighed food records together with an in-person interview approach constitute the most basic methods used to estimate energy and nutrient intakes in dietary surveys. In the background of the coronavirus disease-2019 pandemic, the need for non-face-to-face dietary surveys using information and communication technology (ICT) is increasing. We aimed to evaluate ICT-based dietary record surveys and identify factors that may enable this survey method to become more widely used in the future. We conducted a non-face-to-face survey of dietary records of 44 Japanese individuals, maintained by dietitians using dietary photography and video conferencing services. We conducted a focus group interview with the six dietitians who conducted that survey. Their opinions on the factors necessary to popularize ICT-based dietary survey method were analyzed. In the focus group interview, dietitians highlighted fewer restrictions on time and place as positive aspects. Negative aspects included insufficient skills to operate computers, difficulty in hearing, and understanding facial expressions using ICT. We identified three main factors for enabling widespread use of ICT-based dietary record survey: individual skill, device and technology, and social environmental factors. This suggests that a comprehensive approach is necessary for popularizing the use of ICT in dietary surveys.

## 1. Introduction

Improving the estimation of dietary intakes is essential for evaluating the relationship between health and nutrition. For this purpose, dietary surveys are required to assess the status of the food intakes accurately. To evaluate the dietary intake of a population in relation to specific dietary recommendations, quantified estimates of the dietary intakes may be required [1]. Weighing and recording food portions, one of the dietary record survey methods, includes measuring the weight and volume of the portion sizes using a kitchen scale and keeping a record of the same. It is the most basic methodology used for individual dietary assessment and to ascertain dietary nutritional state [2,3]. The dietary record method formed a part of the National Diet and Nutrition Survey conducted in the United Kingdom until 2019. In Japan, the National Health and Nutrition Survey (NHNS) [4], conducted annually, uses the weighed record method, and the basic process to confirm the weight of food consumed is to conduct face-to-face interviews by trained dietitians and/or registered dietitians. Recently, Zeevi et al. reported high variability of week-long glucose levels in the response to identical meals in a cohort, suggesting that universal dietary recommendations may have limited utility [5]. They devised a machine-learning algorithm that integrates blood parameters and dietary habits, etc., measured in this cohort and showed that it accurately predicts personalized postprandial glycemic response to real-life meals. These reports suggested a need for personalized nutrition by prediction of glycemic responses, which means it is important to grasp the exact diet of the individual. For that purpose, it is necessary to conduct surveys with high accuracy [6].

Globally, there has been a dynamic growth in the use of technology. Over the past decade, technological progress and a significant increase in internet usage has resulted in the development of a number of innovative technologies relevant to dietary assessment. Different technological tools used to address the challenges associated with dietary assessment include mobile food records [7], web-based 24 h recalls [8,9], or food frequency questionnaires (FFQ) [10,11]. Some of these tools are used in national nutritional surveys in the USA and Europe. Self-monitoring of diet or nutritional epidemiology surveys using new technologies, including information and communication technology (ICT), may prove to be a practical alternative to the traditional paper-based systems in the future. In Japan, 82.9% of the population had used the internet in the last one year [12]. Smartphone usage has grown, with 74.3% of the population owning a smartphone, and the rate of internet usage via smartphone is 68.3%, which is the highest usage rate compared to those of personal computers (PCs) and other tablet terminals [12]. In Japan, self-monitoring of diet using new technologies such as smartphone is gaining traction [13,14]. However, these technologies have not yet been deemed suitable for large-scale nutritional epidemiology surveys.

In recent years, the coronavirus disease-2019 (COVID-19) pandemic has intensified the need for non-face-to-face dietary surveys and nutritional guidance using ICT. However, the use of ICT in the field of nutritional epidemiology survey has not been sufficiently explored in Japan yet. The 2020 and 2021 NHNS had to be cancelled due to the spread of COVID-19 in Japan. Since the NHNS is a nutritional epidemiological survey targeting nearly 15,000 people in Japan [4], the practical difficulties in conducting face-to-face surveys and/or interviews during the pandemic was one of the reasons for the suspension of the survey. Therefore, it is urgent to grasp the factors necessary for and problems associated with the widespread dissemination of ICT-based dietary surveys and conducting those surveys in the near future.

The purpose of this study is to evaluate ICT-based dietary record surveys that involved a non-face-to-face interview and to clarify the factors that will enable the widespread adoption of this survey method in the future based on data from focus group interviews (FGI) with participating dietitians who conducted the survey.

## 2. Materials and Methods

### 2.1. Participants in the Nutrition Survey

The nutrition survey, themed “Development of dietary balance optimization technology by estimating dietary intake using biomarkers”, was conducted in January 2022 in Japan. In consideration of COVID-19 control measures, participants were recruited online via e-mail magazines issued by Health Care Systems Co., Ltd. from October 2021 to December 2022. Participants who met the following inclusion criteria were eligible: (1) Japanese nationals aged 20–80 years (at the time of providing informed consent); and (2) those who did not have serious cerebrovascular disease, heart disease, liver disease, kidney disease, gastrointestinal disease, or infectious diseases. The exclusion criteria were as follows: (1) women who were or possible to be pregnant or breastfeeding; (2) those requiring continuous treatment, medication, or lifestyle guidance because of disease; (3) those who were involved in other clinical trials; and (4) those who were judged as unsuitable for participation by the research director.

Six female dietitians aged 43–60 years conducted non-face-to-face interviews with a total of 44 individuals (5 men and 39 women) aged 28–71 years (average age 48.6 ± 10.0 years) regarding their dietary records. This was an observational study, which included a dietary survey component and was conducted by the National Institutes of Biomedical Innovation, Health and Nutrition; National Institutes of Health and Nutrition; Healthcare Systems Co., Ltd.; and Institute of Food Research, National Agriculture and Food Research Organization (NARO).

A semi-structured group interview was conducted by the dietitians and/or registered dietitians (dietitians). The dietitians were recruited from the National Institutes of Biomedical Innovation, Health and Nutrition and other facilities in Tokyo through snowball sampling. Eligibility criteria included the following: (1) dietitians aged 20 years or older and (2) dietitians with experience conducting non-face-to-face interview surveys of dietary records. Based on a previous report, which recommended a group of six to eight participants for FGIs, we recruited and conducted six participants [15,16].

All participants and participating dietitians gave their informed consent before they participated in either the nutrition survey or the FGI. Ethical approval for the present study was granted from the Human Research Committees of the National Institutes of Biomedical Innovation, Health and Nutrition and other facilities, and the research was performed in accordance with the guidelines of the Declaration of Helsinki. (Approval number: Ken-ei 168; Approval date: 2 December 2021. Approval number: Ken-ei 182; Approval date: 12 May 2022).

### 2.2. Dietary Record Survey Utilizing ICT

#### 2.2.1. Dietary Record Survey

Dietary intake data were collected using a two-day, semi-weighed household dietary record, excluding Sundays and public holidays, in January 2022. It was conducted in accordance with the methods of the nutrition survey from the NHNS in Japan [4].

Prior to the nutrition survey, detailed manuals on dietary surveys were distributed to participants, and they were asked to follow the method specified for that dietary survey. Kitchen scales (digital cooking scale KJ-212, TANITA, Co., Ltd., Tokyo, Japan) were handed out to all participants to record the weight of all food consumed over the span of two days. The participants were given detailed instructions to weigh all foods and beverages consumed as well as to note the amount of food waste and leftovers. They were further instructed to record their names and weights on the recording forms. If weighing was not possible (such as in cases where the meal was consumed away from home) the portion size consumed or the quantity of foods and details of any leftovers were estimated. An official food item booklet with standard portion sizes for frequently consumed dishes was applied for estimation conducted by trained dietitians.

All meals consumed were photographed by participants’ smartphones. In order to standardize meal photos, a manual for photographing the meals was created, and the participants were asked to follow these instructions: (1) place a “meal card (4 types: breakfast, lunch, supper, and snack) of dimension similar to that of a business card (55 mm × 91 mm)” next to the meal, and photograph the meal before consumption; (2) photograph the meal at an angle of 90° so that everything you eat in one meal fits in one photo; (3) photos of meals should be taken in sharp focus so that the whole can be seen clearly; (4) be careful not to disclose personal information (employee ID, documents, etc.); and (5) if product name, ingredients, and nutritional labeling of processed foods and seasonings are known, take a photo.

#### 2.2.2. Non-Face-to-Face Interview Regarding Dietary Record Survey

After the paper-based dietary records were mailed to the dietitians by the participants, they were scanned and digitized as PDF files. The meal photos were uploaded to the LINE mobile application (LINE Co., Ltd., Tokyo, Japan) by the participants. Meal photo file names were given by investigators so that the IDs of the participants, the date of meal consumption, and the type of meal (breakfast, lunch, supper, snack) could be distinguished by the dietitians. Google Cloud (Google LLC, CA, USA) was used to share the PDF files of the diet records and meal photos among the meal investigators.

The non-face-to-face interviews regarding the dietary records were conducted by the dietitian concerned using the web-based meeting system Zoom (Zoom, Zoom Video Communications, Inc., San Jose, CA, USA). The dietitians received 5 h of pre-interview training on how to use Zoom and conducted the non-face-to-face interviews on a PC using the network of National Institutes of Biomedical Innovation, Health and Nutrition for the interview survey.

At the start of the interview about dietary records, consent was received from the participants on the following two points: (1) changing the automatically displayed name of the participants on the Zoom screen to their respective IDs and (2) recording a video of the interview to serve as research material. The dietitians checked the dietary records and meal photos before the interview. The Zoom meeting was then started with those materials queued on the PC, which were shared in the course of the interview as appropriate. Information on the contents of dietary surveys, especially on food types, volumes, weights, lengths, etc., were collected in accordance with the interview manual. The dietary record was prepared in both printed paper and digital file formats; printed paper was used for memo-taking during interviews. The interview was generally conducted with the faces of both the nutrition survey participant and the interviewer (dietitian) visible. If the participants refused to turn on the camera, or the camera could not be used on the participant’s system, the interview was carried out without the participant’s face visible. Interviews were conducted for approximately 30–40 min per a participant, and all Zoom meetings were set up at 60 min intervals. The interviewing dietitians conducted the interviews wearing headsets.

In accordance with the survey manual, the trained dietitians converted these estimates of portion sizes or quantity of foods into weights of foods and coded each food item, according to the food number lists based on the Standard Tables of Food Composition in Japan [17] to calculate the intake of energy and nutrients.

### 2.3. FGI

#### 2.3.1. Data Collection from FGI

FGIs are the preferred method for researching a new topic, such as the introduction of new science and technology, where an understanding of the core issues has yet to be developed [15,16]. Moreover, FGIs are a tried and tested method for the exploration, planning, and evaluation of health promotion and nutrition interventions [18]. In FGIs, the type and range of data generated through the social interactions of the groups are often deeper and richer than those obtained from one-on-one interviews [15,16]. This research used a qualitative method, and consideration was given to increase the reliability and validity of the research by conducting it according to the analysis process defined by previous studies [15,16]. Specifically, this involved clarifying the eligibility criterion for participants, the selection process and characteristics of the participants, the interview method, and the FGI analysis process.

Semi-structured interviews of the participating dietitians were conducted in June 2022 for two hours according to the interview guide (Table 1). The researcher (YT) conducted the interview, and another researcher (AY) was in charge of observing the facial expressions and non-verbal reactions of the dietitians. The interviewer (YT) had previous experiences of listening to the dietary records of research in randomized controlled trials and observational studies, and has interviewed patients (nutrition counseling, etc.) as a registered dietitian.

Before conducting the FGI, the researcher (YT) explained the purpose of the study and obtained informed consent from the participating dietitians. The interview was conducted in person with five of the participating dietitians; however, one participating dietitian was interviewed remotely via Zoom (Zoom Video Communications, Inc., San Jose, CA, USA). The interview was recorded by Zoom with the consent of the participating dietitians. The interview was recorded by installing two PCs connected to Zoom in the room, and the facial expressions of the dietitians were recorded from two directions. In order to organize information without omission, the observing researcher (AY) recorded the minutiae of the group interview, such as changes in the facial expressions of each dietitian and subtle gestures such as nodding, from an inconspicuous place. Numbers were used instead of the dietitians’ names during the interview.

All participating dietitians were acquainted with the interviewer. In order to create an atmosphere conducive to easy conversation with the dietitians, the questions at the beginning did not ask for their opinions but asked them to describe their past experiences of interviewing the participants of prior nutrition surveys. Ensuing questions dealt with free remarks. The interviewer proceeded to address the subsequent topics while summarizing the interaction thus far.

The interviews yielded data on what factors, such as equipment, knowledge, support, etc., are needed to enable widespread ICT-based dietary record surveys. Furthermore, the interviews also highlighted problems that may occur while conducting ICT-based nutrition surveys.

#### 2.3.2. Data Analyses

The video recorded on Zoom was converted and transcribed into text-based data to create a verbatim record, and the reactions of the participating dietitians from the observation record were taken into consideration. The transcribed texts were analyzed using qualitative content analysis [16,19]. Upon the obtained data, coding and categorization were performed using a content analysis method. Two authors (YT and AT) condensed the text and then abstracted and labeled it using a code, considering the text as a whole. The codes were compared based on their differences and similarities, and they were then sorted into sub-categories under individual categories. Authors analyzed and reflected on the analysis process and the coding frame. Quotes from the interviews are presented in Section 3 to illustrate the categories and sub-categories. Qualitative data analysis software (NVivo Windows Release 1.6.1, QSR International Pty Ltd., Melbourne, VIC, Australia) was used for coding and categorization.

## 3. Results

### 3.1. Participating Dietitians’ Characteristics, Experiences of Interview in Nutrition Survey, and Non-Face-to-Face Meeting in Web

The characteristics and interview experiences of the six female dietitians who participated in the FGI are presented in Table 2. The average age of the dietitians was 51.3 ± 6.1 years. The dietitians’ experiences ranged from 3 to 15 years of conducting face-to-face interviews, with 6–140 people, predominantly at public health centers, etc. In terms of non-face-to-face experience, the number of people with whom the dietitians conducted web-based nutritional interviews ranged from 1 to 11 people, while the frequency of remote interactions, excluding dietary surveys, ranged from 5 to 100+ times. The devices and applications used for web-based meetings were PCs and smartphones and Zoom, LINE, WebEX (Cisco Webex, Cisco Systems, Inc., San Francisco, CA, USA), Microsoft Teams (Microsoft Corporation, Redmond, WA, USA), and Skype (Microsoft Corporation, Redmond, WA, USA).

### 3.2. Analysis of FGIs

#### 3.2.1. Positive Aspects and Negative Aspects on Non-Face-to-Face Dietary Surveys

We first classified the data extracted from FGIs into positive and negative aspects of non-face-to-face interviews for checking dietary records. The positive aspects were further sub-divided into four categories: less time and place restrictions; easy confirmation of seasonings and foods; photos and diet records can be shared between dietitians and participants; and the relaxed atmosphere allowing participants to easily recall what they ate. Representative codes are shown below:

*“Participants did not have to go out and could get interviewed at home or at work, so I think it has led to saving their time.”*

(#2)

*“I was able to check through Zoom the seasoning, foods, and/or food labels of what the participants actually ate.”*

(#6)

*“It was great that we were able to share food photos and diet records between interviewer and participants, and it was also easy for the participants to recall what they ate.”*

(#5)

*“Participants felt relaxed when they were being interviewed at home, so it was easy to hear various factors related to their diet.”*

(#3)

Further, the negative aspects of non-face-to-face dietary surveys were sub-divided into three categories: difficulty in conversation and using machine and tools due to lack of requisite skills; difficulty in listening to the sound and/or understanding participants’ facial expressions on screen due to the participant’s interview location and/or equipment used; photos that could not allow dieticians to ascertain the type of meal intake, foods, and their weights and size. Representative codes are shown below:

*“It was difficult to do…, maybe it was a computer skill problem on my part, and I was unfamiliar with it.”*

(#1)

*“I felt that smartphones are not suitable for conducting interviews of dietary records because it was a bit difficult to talk while understanding the participants’ expression”*

(#3)

*“The participant was being interviewed while travelling in a car, so I asked her to stop the car, and then proceeded with the interview.”*

(#4)

*“There were photos that looked like they featured just a pot with raw food ingredients, and it was a bit hard to judge how foods were prepared and how they were consumed.”*

(#5)

The summaries and examples of responses to the three major FGI questions (relating to factors that require careful attention, useful tools, and note-taking methods during non-face-to-face nutritional interviews) are shown in Table 3.

#### 3.2.2. Factors for Widespread Use of a Dietary Record Survey Utilizing ICT

**Figure 1:**
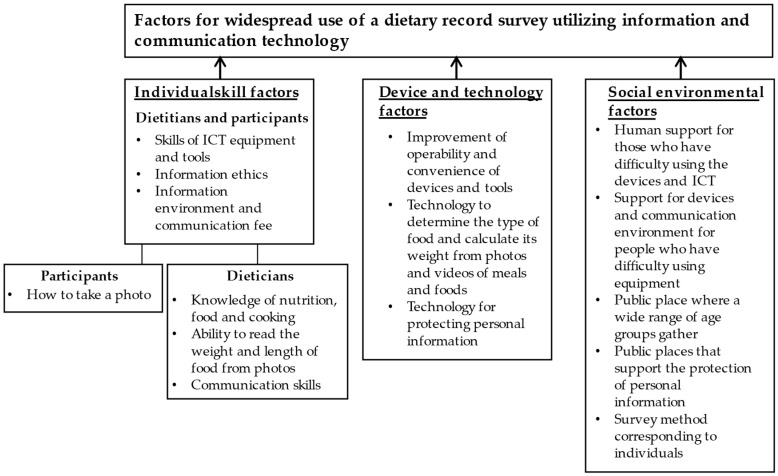
Categories and sub-categories of factors for widespread use of a dietary record survey utilizing information and communication technology (ICT) using focus group interview.

From the FGIs, we identified three main categories of factors: individual skill, device and technology, and social environmental factors, as summarized in Figure 1.

Individual skill factors: We classified individual skill factors into three sub-categories: those of both the dietitian and participant; those of the dietitian; and those of the participant. Factors were classified into sub-categories within each type. Individual skill factors among both dietitians and participants were divided into the following categories: ICT skills (equipment and tools), information ethics, information environment, and communication fee. Representative codes are shown below:

*“I think that if I had more knowledge about computers, I could do more things.”*

(#4, 5)

*“I think there are quite a few people who are not conscious of personal information”*

(#1)

*“If there is no Wi-Fi environment, communication charges will be incurred”*

(#2)

Individual skill factors among dietitians were further divided into the following sub-categories: knowledge of nutrition, food, and cooking; ability to assess the weight and size of food from photos; and communication skills. Representative codes are shown below:

*“If I could imagine the meal that participants ate, and/or had the knowledge of the food products …I felt that I had very few questions for participants.”*

(#6)

*“If as soon as I saw meals and foods, I could imagine what ingredients were used, and what the salt absorption rate of the dish would be, and what the weight conversion would be, I will be able to estimate nutritional values easy for dishes*

(#6)

*“I spoke more clearly during the interview because it was over the screen.”*

(#5)

*“I was careful to have the conversation calmly and with a smile.”*

(#1)

Individual skill factors among participants were related to how to take a photo.

*“It’s nice to have a photo, but sometimes I could not estimate when the meal was eaten, or the content and amount of the meal the participants ate from the meal photos...”*

(#5)

Device and technology factors: We classified device and technology factors divided into the following sub-categories: improvement of operability and convenience of devices and tools; technology to determine the type of food and calculate its weight from photos and videos of meals and foods; and technology for protecting personal information. Representative codes are shown below:

*“Participants would like to use simpler and easy-to-use applications, such as using those assisted with artificial intelligence (AI) etc.”*

(#3, 5)

*“For example, it would be nice if it could be as simple as just taking a photo, the smartphone application can estimate the type of meal and foods, and their weights, etc.”*

(#1, 6)

*“I would like to have a machine that, just by taking a video of the person making the meal, could estimate what ingredients were used and how much spices were added”*

(#2)

*“If the accuracy of weight estimation for foods is improved, there is less need for detailed interviews, so I think it’ll be okay then if you can’t see the face”*

(#6)

*“I would like to have an application that once you launch the tool, PC or application will recognize things that move, focusing only on that part, and blurring out other parts.”*

(#5)

Social environmental factors: The social environmental factors were sub-divided into five sub-categories: human support for those who have difficulty using the devices and ICT; support for devices and communication environment for people who have difficulty using the equipment; public places where a wide range of age groups gather; public places that support the protection of personal information; and survey method corresponding to individuals. Representative codes are shown below:

*“It would be nice if there was someone in public who could teach me about PC, applications, and ICT.”*

(#1)

*“Even if participants had a smartphone, depending on the conditions of the contract, they may not be able to respond without a Wi-Fi environment...”*

(#3)

*“If it was possible to easily conduct dietary record surveys using tablets, I think many people would take nutrition surveys.”*

(#5)

*“I think it would be better to do it together in a place like a public gathering place with 4 to 5 people”*

(#2)

*“Places where personal information is protected and the network can be used in public are needed… example, it may be a railway station, etc.”*

(#5)

## 4. Discussion

This study focused on the challenges in using ICT in large-scale nutritional epidemiological study such as NHNS in Japan in the future. The data of NHNS are used to monitor diet and nutritional intake at a population level in order to provide the evidence base for developing and evaluating health policy. The utilization of ICT in a nutrition survey is considered to be an important issue for future nutrition policy.

As reported previously, internet access is constantly increasing throughout developed countries and across age groups [20]. Thus, the advantages of using ICT are irrefutable. A dietitian-led interview is commonly used as a “gold standard” to evaluate the validity of self-administered tools. There were some positive aspects in the non-face-to-face interviews conducted as part of our study. First positive aspect was reduced time and place restrictions. In this nutrition survey, although many study participants were interviewed from their homes, there were a few who participated from their workplace. Being able to take the survey in a short time without moving to a specific location may reduce the burden and constraint for the participant. Secondly, the dietitian can directly ask the participant when a mistake in the declared type and quantities of foods is suspected, thus avoiding errors in potentially difficult portion size estimates. Moreover, dietitians could ask the participants to bring the specific foods in front of the camera and were able to check the package and nutrition labels of foods through Zoom. Moreover, it was easy to check the meals’ content and their weight by being able to share and check the dietary records and meal photos on the screen together.

In contrast, the non-face-to-face interview also had several negative aspects. Firstly, it was difficult for the dietitian to respond when something unexpected happened to the PC and their tools due to the lack of skill with machines and tools. In this non-face-to-face interview, indeed, we had PC and tool troubles, such as not being able to hear the participant’s voice. Next, it is difficult to understand the face and facial expressions when the participant is using a smartphone, and when the participant was not wearing a headset, and the ambient sound made it difficult to hear the participant’s voice. Previous studies have also reported that in the case of ICT methods using web meeting systems, it is sometimes difficult to hear conversations, depending on the system conditions [21]. In some cases, it may be necessary to ask not only the dietitian but also participants to wear headsets in advance. Therefore, the non-face-to-face interview method using ICT for dietary records in nutrition survey has both positive and negative aspects compared with the face-to-face interview method.

In the FGI of participating dietitians, “knowledge of nutrition, food, and cooking”, “ability to assess the weight and size of food portions from photos”, and “how to take photos” as individual skill factors and “technology to determine the type of food and calculate its weight from photos and videos of meals and foods” as device and technology factors were cited as the most crucial factors for the spread of dietary survey methods using ICT. In other words, it can be said that determining the type of food and estimating its weight from food photographs is a very important process in nutrition research. Photos of a participant’s dietary intake in a nutrition survey have been used to assist traditional dietary assessment methods for portion size estimations by dietitians (image-assisted methods). Image-based approaches such as meal photos aimed at capturing all meals consumed and images as the primary record of dietary intake follow the methodology of food records. Therefore, image-assisted approaches can supplement dietary records.

The development of dietary survey methods using new technologies including ICT can be divided into two types [22]. The first type includes acquisition of simple self-reported dietary data, provided that the method of acquiring self-reported dietary data and new technologies including ICT are included in the interviews. The second method is based on the assumption that the acquisition of meal data itself is automated or semi-automated. In our study, non-face-to-face interviews were conducted with meal photos, but the nutrition survey itself followed the standard method of dietary record using paper and pen by participants. Therefore, our study adheres to the first type. Nutrition surveys using the first stage have previously been reported to be useful [23]. In the first type, the simple pen-and-paper approach is most often replaced with a keyboard or smart phone, etc., but it does not fundamentally change the workload of the participants. The second type includes the use of sensors and AI to automate data entry and reduce the workload. Recently, the second type of methods have also been reported frequently. For example, the technology-assisted dietary assessment system uses the integrated digital camera in a mobile device for taking food photos [24,25,26].

Notably, Stumbo suggested that it is difficult to automatically identify a food and estimate its portion size from photo, and photos on their own usually require additional information, unless the photographing process is standardized [27]. In order to improve the accuracy of image analysis of food photos, the method of photography is important. Xu reported that to enhance the image analysis process, the image is best captured between a 45–60° angle [28,29]. In our interviews as well, the participant’s “How to take a photo” was mentioned as an individual skill factor. In the process of adopting new technologies in nutritional surveys, the method of photographing is likely to be one of the key factors. Some projects (eButton and Technology assisted dietary assessment [24,25,26]) have developed algorithms to use a single image in calculating volume assessment. The eButton wire mesh is a method that attempts to solve the issue of portion size estimation. The authors found good reliability and validity upon comparing size estimations. In other research, an electronic dietary intake assessment application was developed for use as a weighted or estimated food record and tested against repeated 24 h recalls [30]. The results showed that a large variability of reported intakes at the individual level was observed. Nutritional assessment methods that incorporate these new techniques are of limited use in large-scale epidemiological studies, and further investigations are required in the future.

Social environmental factors were cited as factors for the spread of dietary survey methods using ICT; specifically, they were human support, devices and communication environment, and public space. Increasing mobile phone and internet access are not a panacea, and there were concerns that acceptability of new technologies might be low among some sections of the population (even those with access), mainly for those who were not skillful, well-versed, or familiar with technology. In a previous study using web-based, self-administered 24 h dietary records of middle-aged participants, seven participants stopped because they felt the web system was too complex for them [20]. In field testing of INTAKE24, low response rates among older people were reported [31]. In the FGI in this study, there were many concerns about people who have difficulty using machines and tools with ICT. The internet usage rate of the entire population in Japan was 82.9%; however, the same for those in their 70s was 59.4%, and for those in their 80s was 27.6%. These rates were much lower than other generations. In our study, 44 participants participated in the non-face-to face dietary survey, and the average age was 48.6 ± 10.0 years. Additionally, the participants were recruited through the e-mail magazines; therefore, they may not necessarily be representative of a standard Japanese population with regard to ICT skills. These reports suggest that skill support in using devices, such as PCs and tools using ICT, for the elderly and those not skilled, well-versed, or familiar with technology is also necessary in Japan.

Moreover, since online surveys can only be used by people who can use communication devices and ICT, there is a possibility that the population sample would be biased. It has been suggested that the quality of computer-based assessment may decrease with age [32] and increase with educational level and computer literacy [33]. Meanwhile, population groups with variable cognitive skills and computer literacy (e.g., young children, older adults, and non-technology users) could perhaps benefit more from interviewer input, either by face-to-face or telephonic support [31,34]. In our FGI, “skills of ICT equipment and tools” was cited as a factor necessary for the widespread use of a dietary record survey utilizing ICT by both dietitians and participants. Therefore, there may be a need for a public support system for computer literacy and their usage environment, not only for the target participant but also for the dietitians as interviewers in nutrition survey. These suggests the importance of carefully considering the implementation of new technologies into nutrition surveys and the potential need for tailored support for study participants and dietitians.

In dietary surveys, there is concern that personal information is reflected in photographs and interviews. In our interview, the personal information concern was emphasized in “Device and technology factors” and “Social environmental factors”. The Japanese government report on information also discusses concerns about information morals such as the protection of personal information [12]. Research on how to address privacy concerns and ensure full compliance with the General Data Protection Regulation in automatizing dietary intake data collection has been reported [22,35]. AI-based algorithms which can automatically detect food items from images acquired by a wearable camera for dietary assessment have been developing over time [36]. The results suggest that the approach has the potential to automatically identify foods from low-quality, wearable camera-acquired images with reasonable accuracy. Although the research in turn seems to be able to reduce both the burden of data processing and privacy concerns, the estimation of weight is an issue if results are to be translated into nutritional values.

Currently, research on new technologies including ICT is ongoing in national nutritional epidemiological surveys in the United States and European countries (UK and Germany, etc.). To the best of our knowledge, the national nutrition surveys utilizing new technologies such as ICT have not been implemented in nutrition and health surveys in Asian countries, including Japan, yet. In the future, it may be necessary to consider specific survey methods that utilize ICT and new technologies for NHNS in Japan.

In the 1990s, along with the development of science and technology, a scientific field called nutrigenomics was also developed. Since the 2010s, numerous association studies have been performed to identify genetic factors that may explain individual differences in metabolic responses to specific diets, indicating that individuals with different alleles are affected in different way by the same nutrient intake [37,38,39]. These studies highlighted the need for personalized nutrition, which links detailed information related to the individual, including nutrient intake, genotype, microbiota, metabolism, and physical activity, with health. Based on this history, as with other factors that constitute precision nutrition, nutrition assessment has also supported precision nutrition against the background of technological developments such as ICT. Since the 2000s, many research approaches for nutrition surveys and assessments using ICT have been developed [40,41,42]. In 2009, Ngo et al. conducted a systematic review of ICT for nutrition assessment and reported that non-face-to-face computerized assessments (FFQs, 24 h recalls, and diet histories) and personal digital assistant (PDA) assessments (PDA-based food records, PDAs with camera and mobile phone card, digital photography) were promising and could improve dietary assessment quality in some vulnerable groups, though comprehensive evaluation for micronutrient intake assessment was still needed [43]. In 2013, the Food4Me project based in seven European countries aimed to capitalize on improved internet access to evaluate an online precision dietary intervention, and habitual dietary intake data were collected and quantified via web-FFQ [11,44]. In the future, to investigate detailed nutrient intake amounts, it may be useful to conduct dietary non-face-to-face interviews in addition to such methods to double-check dietary information submitted through ICT.

There are certain limitations of this study. First, the FGI was conducted with a small number of participating dietitians from one institute. Our results may still be biased because of the small sample size and the limited recruitment process. This could limit the generalizability of the present study. Second, only one dietitian participated in the FGI remotely; the other dietitians participated in the face-to-face interview in the same room. Because the characteristics and circumstances of one dietitian was different from other dietitians, the group dynamics may have been affected. Third, one participating dietician had only one experience of non-face-to-face interviewing. However, she also managed the dieticians and their interviews and supported the other dietitians during their non-face-to-face interviews. Therefore, she was able to gain good insight into the logistics and problems of non-face-to-face interviews for dietary surveys and was deemed suitable as a participating dietitian for our FGI.

## 5. Conclusions

This study highlights the challenges in using ICT-based dietary records method in nutritional surveys. We identified three main categories of enabling factors for the widespread use of an ICT-based dietary record survey: individual skill, device and technology, and social environmental factors. These observations suggest that a comprehensive approach is necessary for popularizing the use of ICT for large-scale nutritional epidemiological and dietary surveys in the future.

## Figures and Tables

**Table 1:** Focus group interview outline.

Line of Inquiry	Questions
The experience of an interview at Nutrition Survey	1. The experience of a face-to-face interview at Nutrition Survey
	2. The experience of a non-face-to-face interview at Nutrition Survey
	3. What type of the machines and tools used in the interview
How to handle a situation in a non-face-to-face interview at Nutrition Survey	4. Preparations for non-face-to-face interviews and their time
	5. How were machines and ICT used for non-face-to-face interviews?
	6. Useful devices and tools for non-face-to-face interviews
	7. Necessary skills to use information communication devices (ICT) and tools for non-face-to-face interviews
	8. How voice was heard and whether headsets are used in non-face-to-face interview
	9. Tools used to confirm the weight and size of food and points to note in non-face-to-face interviews
	10. Functions of the PC used to confirm the weight, size, and type of food in non-face-to-face interviews
	11. How to take notes during non-face-to-face interviews
	12. Other points to note in non-face-to-face interviews
Positive and negative points in non-face-to-face interview at Nutrition Survey	13. Positive points
	14. Negative points
Factors for widespread use of a dietary record survey utilizing ICT	15. Machines
	16. Knowledge and Skills
	17. Problems that may arise

**Table 2:** Characteristics and interview experience of participating dietitians.

	Participating Dietitians					
ID	#1	#2	#3	#4	#5	#6
Sex	Female	Female	Female	Female	Female	Female
Age (years)	58	46	52	60	43	49
Experience of conducting face-to-face nutritional interviews						
Year(s)	15	3	7	3	5	14
Number of persons	7	15	140	70	20	100<
Place	NHNS *^1^, public health center	NHNS *^1^, public health center	NHNS *^1^, research institute	Hospital, research institute	Facility for the elderly	Research institute, public health center
Experience of conducting non-face-to-face/remote nutritional interviews						
Number of persons	10 *^2^	8 *^2^	8 *^2^	11 *^2^	1 *^2^	6 *^2^
Place	Research institute	Research institute	Research institute	Research institute	Research institute	Research institute
Other experiences of non-face-to-face/remote interactions						
Type	Study meetings	Study meetings, meeting friends and/or colleagues	Study meeting, workshop	Study meeting, workshop	Study meeting, workshop, meeting friends and/or colleagues	Cooking class, study meeting, workshop, meeting friends and/or colleagues
Number of times	30	20–30	20–30	5–6	>100	30–40
Device	PC *^3^, smartphone	PC *^3^, smartphone	Smartphone	PC *^3^, smartphone	PC *^3^, smartphone	PC *^3^, smartphone
Application	Zoom	Zoom, LINE	Zoom, LINE	Zoom, LINE	Zoom, WebEx, Teams, Skype, LINE	Zoom, LINE

*^1^ National Health and Nutrition Survey; *^2^ in our study; *^3^ personal computer.

**Table 3:** Summaries and examples of responses to major FGI questions.

Themes	Summary	Examples
To what factors did you pay attention in the non-face-to-face interviews of Nutrition Survey?	Listening attentively in stipulated time	I was careful to finish the session within 30 min to a maximum of 1 h. (#2, 5)/I paid attention to ask simple questions. (#3)
	Missing entries in the dietary records	I tried not to forget to ask about the types of seasonings the participants used or what they were eating that wasn’t in the photos, to generate comprehensive dietary records. (#2)
	Using louder voice and exaggerated gestures to make it easier to convey my message.	I spoke more clearly because the message had to be conveyed through a screen. (#5)/I tried to use gestures such as exaggerated nodding. (#5)
	Create an atmosphere that is conducive to conversation	I was careful to be gentle in proceeding. (#6)/I chose clothes with bright colors to brighten the impression of the screen. (#1)
Useful tool in non-face-to-face interviews at Nutrition Survey.	Tools for information communication equipment (ICT)	I was wearing a headset, so my voice didn’t overlap, which was good. (#2)/Meal photos and dietary records can be shared through PC screen, and could be confirmed with the target person, which was very useful. (#5)
	Meal card	It was easy to imagine the size of the meal and the food portions with the meal card, which is the size of a business card, present alongside the meal in photos. (#3) The photograph of the meal card together with the meal made it easy to grasp the day on which the meal was eaten and the classification of meal as breakfast, lunch, dinner, or snacks. (#4)
	Weighing instruments/tableware	I’ve actually used measuring spoons, plates, and ruler during interviews at Nutrition Survey. (#3)
	Hand	I verified the size by sticking out my hand and asking, “Is it about this size?” (#1)
How did you take notes during the non-face-to-face interviews at Nutrition Survey.	Filled in the Nutrition dietary records (paper) with a pen	Using an erasable pen, I wrote down what I heard in the interview on the right half of the meal record sheet filled in by the participants, in a rather crude manner at that time. After the interview, I used an erasable pen to take notes so that I could rewrite them. (#2)
	Appropriate use of screenshots	Those who participated in the survey from their homes brought seasonings in front of the PC and when the participants showed me the seasonings through the camera, I took a screenshot of the same on my computer and looked at the contents later in greater detail. (#2, 6)
